# Pigeon in a Box: *Columba livia* as Subject in Behavioral Research

**DOI:** 10.1007/s40614-025-00454-4

**Published:** 2025-05-29

**Authors:** Eduardo J. Fernandez, Kennon A. Lattal

**Affiliations:** 1 School of Animal and Veterinary Sciences, University of Adelaide, Adelaide, SA 5005 Australia; 2 Department of Psychology, West Virginia University, Morgantown, WV USA

**Keywords:** Behavior analysis, Experimental analysis of behavior, Laboratory research, Pigeon, *Columba livia*

## Abstract

Pigeons (*Columba livia*) have played a central role as subjects in the experimental analysis of behavior since the 1940s. This review considers the use of pigeons by humans across several domains: (1) their early use as a domesticated species and in early psychology laboratory experiments; (2) their rise, and recent decline relative to the use of other species, as a subject in behavior-analytic research published in the *Journal of the Experimental Analysis of Behavior*; and (3) their influence in research extending beyond behavior analysis. In addition, in the latter two sections, quantitative data are presented to document the frequency of use of laboratory pigeons and their impact outside of the lab, respectively. The review concludes with observations on both the past and future of the pigeon as a subject for the experimental analysis of behavior.

Common pigeons (*Columba livia*; see Donegan, 2016) have played a critical role in the development of behavior analysis, (see, e.g., Lattal, 2004; Logue, 2002; Skinner, 1956). It was their initial use as subjects in Skinner’s bomb-guide training, Project Pigeon, in the 1940s (Skinner, 1956) that led to the “discovery” of the differential reinforcement of successive approximations (response shaping; Capshew, 1993; Peterson, 2004; Skinner, 1960). Project Pigeon also was one of the earliest applied endeavors of behavior analysis, as well as inspiration for another early application: animal training (Breland & Breland, 1951; Fernandez & Martin, 2021, 2023; Lattal & Fernandez, 2022; Skinner, 1951). Nonetheless, Skinner’s (1948) “Superstition in the Pigeon” marked their debut in peer-reviewed psychological literature, more than half a decade after their first use in Project Pigeon. In addition, research involving pigeons was seldom published in psychological journals until the establishment of the *Journal of the Experimental Analysis of Behavior* (*JEAB*) in 1958. These developments invite questions about how pigeons went from such modest laboratory-subject beginnings to become one of the most important subjects used in the science of behavior.

This review therefore considers the use of pigeons by humans across several domains: (1) their early use as a domesticated species and in early psychology laboratory experiments; (2) their rise, and recent decline relative to the use of other species, as a subject in behavior-analytic research published in *JEAB*; and (3) their influence in research extending beyond behavior analysis. The review concludes with observations on both the past and future of the pigeon as a subject for the experimental analysis of behavior.

## Pigeon Domestication and Early Laboratory Use

Although *Columba livia* is one of the most widespread domesticated avian species, little has been written about the species’ early domestication history (Gilbert & Shapiro, 2014; Johnston & Janiga, 1995). Levi (1981) traced the domestication of pigeons to 5000–2000 bce, noting also their earliest appearances in art, religion, and other mythological writings. Much of the documented history of the pigeon begins in the mid-nineteenth century, when individuals interested in continued domestication efforts, including Charles Darwin, detailed such events (Bartley, 1992; Secord, 1981). Darwin was particularly interested in the common pigeon as a model of artificial selection, or the process of domestication itself (Darwin, 1859; Theunissen, 2012). Before their (then) new 19th-century role in Darwin’s theory of evolution, pigeons had for centuries served humans in other capacities: food source; targets for training birds of prey and other animals for hunting; entertainment; a reason for wagering over their racing acumen; the namesake of 10 cities worldwide; and, perhaps most famous, as carriers of messages over long and short distances, particularly during times of war (Levi, 1981). Concerning the latter, the leaders of Lille, France, erected a 20-foot-tall monument at the entry to that city’s largest park in remembrance of the 20,000 pigeons that died in World War I and the pigeon fanciers who were executed for keeping them (Lille Tourism, 2024). It was only in 1957 that the U.S. Army Signal Corps disbanded (no pun intended) its pigeon facility at Fort Monmouth, New Jersey (U.S. Department of Agriculture National Agricultural Library, 2025).

Notwithstanding that interest in the domestication of pigeons helped lay the groundwork for later laboratory pigeon use, little psychological research involved pigeons prior to Project Pigeon. In one early published experiment involving pigeons, The prominent learning theorist O. Hobart Mowrer used pigeons in some of his earliest research. His doctoral dissertation research at Johns Hopkins University (Mowrer, 1932), under the supervision of Knight Dunlap, and several subsequent experiments were concerned with the nystagmic response of pigeons (e.g., Dunlap & Mower, 1930; Mowrer, 1935). Hamilton and Coleman (1933) also used a modified version of the Lashley (1930) jumping stand to study color discrimination in that species. Such research, however, appears to have developed independently of and to have had no impact on the use of pigeons in the experimental analysis of behavior (EAB). Rather, the impetus for their use as laboratory animals in EAB came directly from Project Pigeon. The details of that project, wherein between 1941 and 1943 Skinner and colleagues trained pigeons to guide bombs as part of the larger war effort (Capshew, 1993; Peterson, 2004; Skinner, 1956, 1979), are well-known.

The idea for Project Pigeon came when Skinner, on his way by train to the 1940 Midwestern Psychological Association conference in Chicago, observed wild pigeons in flight:

I saw a flock of birds lifting and wheeling in formation as they flew alongside the train. Suddenly I saw them as 'devices' with excellent vision and extraordinary maneuverability. Could they not guide a missile? Was the answer to the problem waiting for me in my own backyard? (Skinner, 1979, p. 241)

**Fig. 1:**
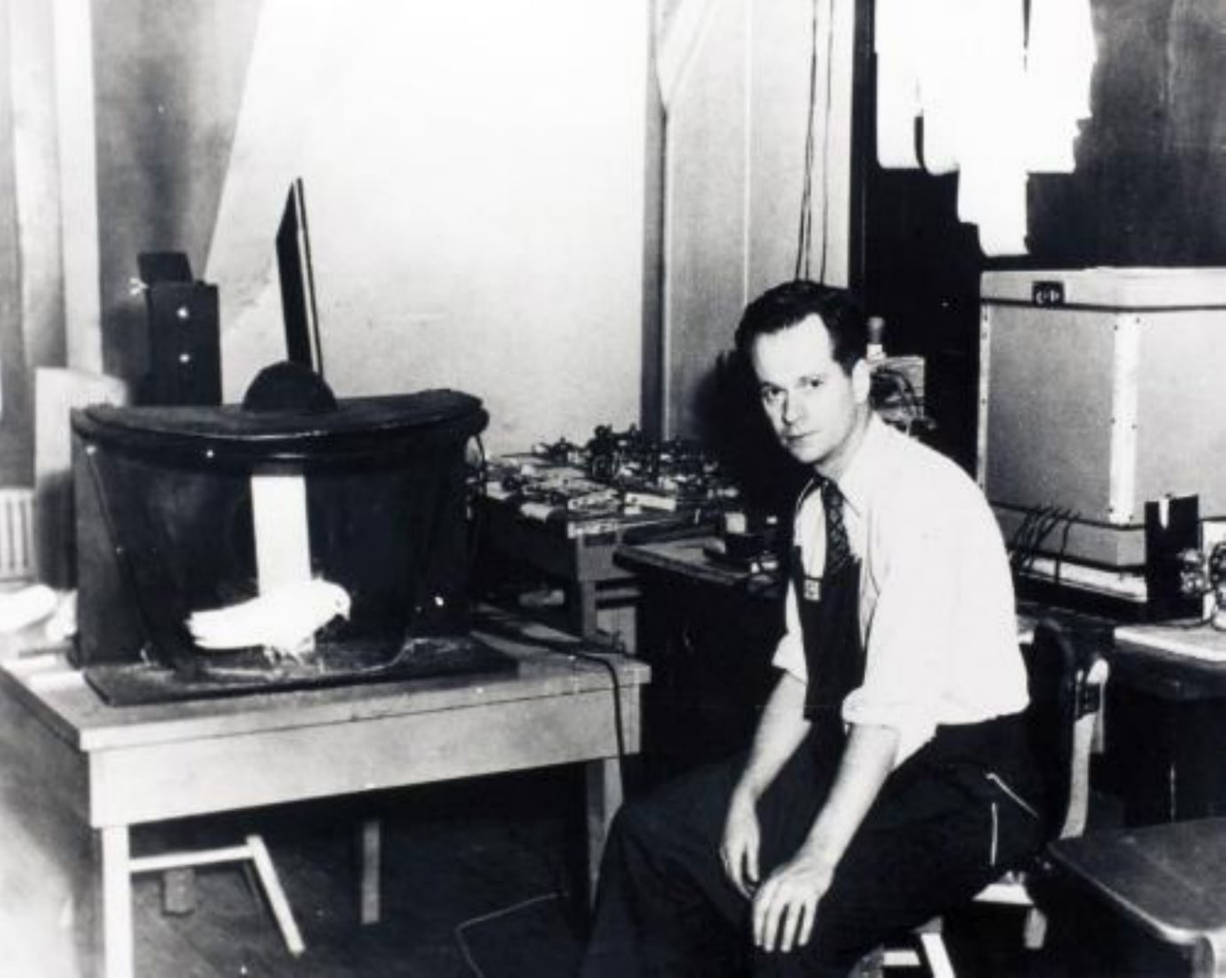
Photograph of B. F. Skinner Working with a Pigeon in an Early Operant Chamber at Indiana University, circa 1947. *Note.* The pigeon may be standing on the tambour mechanisms mentioned in Skinner (1948) for measuring steps. The chamber does not appear to have a key-peck operandum. (Photo courtesy of the B. F. Skinner Foundation)

Although the above marked the first use of pigeons by Skinner in laboratory research, it was several more years before he published any such research (Skinner, 1948). Even then, the data appearing in “Superstition in the Pigeon” consisted of but a single cumulative record, which was the result of recording steps on a tambour-like device (see Fig. 1). He earlier, however, had reported on experiments using pigeons at the first Conference on the Experimental Analysis of Behavior held in June 1947 at Indiana University: “Skinner described some work with concurrent schedules (sometimes using compatible responses and at other times recording from each of two keys), his work on matching [perhaps, matching to sample?], and some exploratory work on the problem of reaction time in the pigeon” (Dinsmoor, 1987, p. 444).

In the above quotation, Skinner (1979, p. 241) provided two reasons why he selected pigeons for subjects in his Project Pelican: their visual system and their maneuverability. Other physical characteristics that made pigeons a valuable subject were their longevity (many live more than 20 years in captivity—the second author of this review had one pigeon in his laboratory for over 30 years) and their generally good health and hardiness, commending them for long-term and multiple experiments over extended time periods. As the pigeon began to be adopted for research, other behavioral characteristics reinforced the selection. Ferster (1953b) observed that, on the one hand, “[t]he pigeon comes to the experimenter with a well-tailored response extremely suitable for free operant type experimentation. The high rates of pecking that can be generated in the pigeon result in a dependent variable which can change over a very wide range, and which is necessarily more sensitive to manipulation” (Ferster, 1953b, p. 271). On the other hand, Ferster also cautioned that “[t]he pecking response would, of course, be extremely unsuitable where variables influencing the form of the behavior were being manipulated” (p. 265). In addition, early on the generality of previous behavioral findings with other species were systematically replicated and expanded with pigeons, further establishing their value as research subjects in the experimental analysis of behavior.

Skinner’s (1948) “superstition” experiment appeared the same year that he returned to Harvard and established what was to be known as the “Pigeon lab” (see Lattal, 2002). Ferster (2002), who was a postdoctoral fellow with Skinner from 1951 to 1955, noted that “[t]he laboratory was in operation when I arrived in Cambridge [MA]. Several graduate students were preparing pigeon demonstrations for Skinner’s introductory course and there were several pigeon boxes with relay control apparatus” (p. 38). Once Skinner started the ball rolling (or, perhaps more apropos to the current discussion, the pigeon pecking), some of his first students began using pigeons (Ferster, 1953a, 1954; Heise, 1953), although it was almost another decade before Skinner published further research involving pigeons as subjects (Ferster & Skinner, 1957; Skinner & Morse, 1957). The early core of “pigeon researchers,” most of whom trained with or were heavily influenced by Skinner, further established the precedent of conducting behavioral research with pigeons, which then expanded exponentially with their moves to universities across the world and the subsequent establishment of *JEAB.* It is to that topic that the review now turns.

## Pigeons in the Experimental Analysis of Behavior

The creation of *JEAB* in 1958 marked the first journal dedicated solely to EAB. That creation has been recounted by Laties (1987). It also became the epicenter of published pigeon research. To better visualize this emergence, it is useful to track pigeons and other species as subjects throughout *JEAB’s* existence. These results are detailed below.

## Method

For each research article published in *JEAB* between 1958 and 2023 (66 years), experimental subjects were sorted into one of five categories: Pigeons, Rats, Humans, Nonhuman Primates, or Other (any taxonomic species other than those that fit in the previous four categories). Only empirical articles were considered, which were defined as publications that used at least one live subject (thus excluding virtual organisms or previously published data) and included some quantitative or graphical description of behavior. In addition, any one article was counted as more than one of the subject categories noted above, when more than one species was used in a publication. For instance, Conrad et al. (1958) used five rats and one monkey as subjects. Therefore, this resulted in the publication being counted as one empirical article incorporating two subject categories.

Interobserver agreement (IOA) was calculated based on total agreement (Poling et al., 1995) for empirical articles and for four of the subject categories (Pigeons, Rats, Humans, and Other [Nonhuman Primates and Other combined]), as well as for 20 of the 66 years, or 30% of all the data collected. Agreement was determined by the first author functioning as the primary observer and comparing 19 of the 20 comparison years (2004–2022) based on data collected by the *JEAB* editorial staff (M. Galizio, personal communication, April 24, 2023). The 20th and final (2023) comparison year was calculated by the second author. All IOA calculations for any one category and year ranged from 50 to 100%, with agreements below 80% only occurring when counts for any one category were below five articles (e.g., two and four articles counted for each observer, producing an agreement of 50%). The final average for each of the categories was > 95% agreement (Empirical Articles: 99.4%; Pigeons: 98.5%; Rats: 97%; Humans: 97.3%; Other: 95.5%).

## Results

**Fig. 2:**
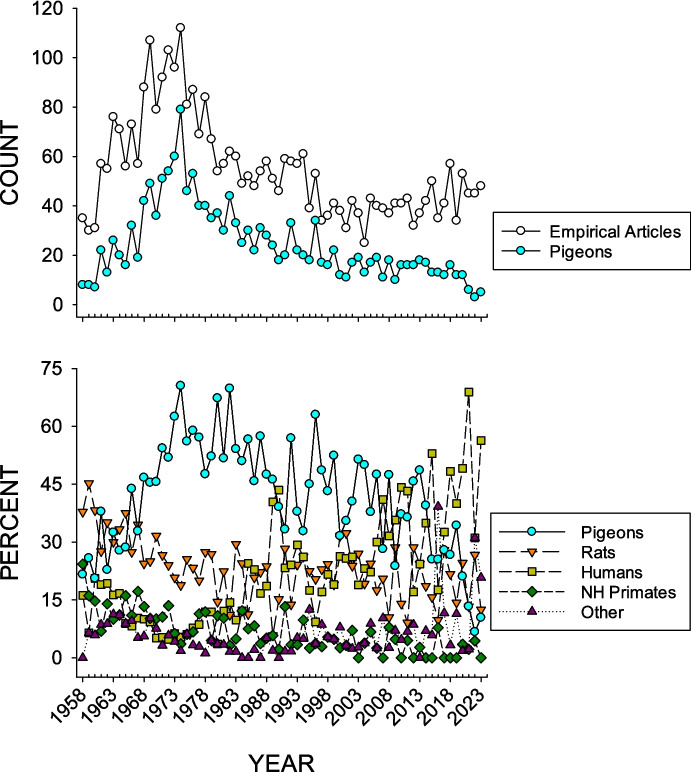
Frequency of Empirical Articles and Pigeon JEAB Publications (Top Graph), as Well as the Percentage of Articles per Each Taxonomic Category (Bottom Graph). *Note.* The x-axis shows the year-to-year values, 1958–2023

Figure 2 shows both the number of articles in which pigeons were used compared to the total number of empirical articles, as well as the percentage of pigeon articles compared to articles in which rats, humans, nonhuman primates, and other species served as subjects, over all of the issues of *JEAB* published from its inception in 1958 through 2023.

During the first decade of JEAB’s existence (1958–1967), rats were typically the most frequently used subjects. The exceptions were 1961, 1963, and 1966, when pigeons were used more often than other species. By 1968, pigeons became the most frequently used subjects, continuing with almost no exceptions (in 1990, however, humans were more commonly used) until 2006. During this time, pigeons reached their most frequent use as subjects in 1974, when they accounted for 70.5% of all publication subjects. From 2007 to 2011, humans became more frequently used subjects for most years, followed by a resurgence in 2012–2014 in pigeon-based research. Since that time (2015–2023), humans have become the most used research subjects, and in the last 2 years, both rats and other species have been more frequently used as subjects in *JEAB* articles than pigeons.

**Fig. 3:**
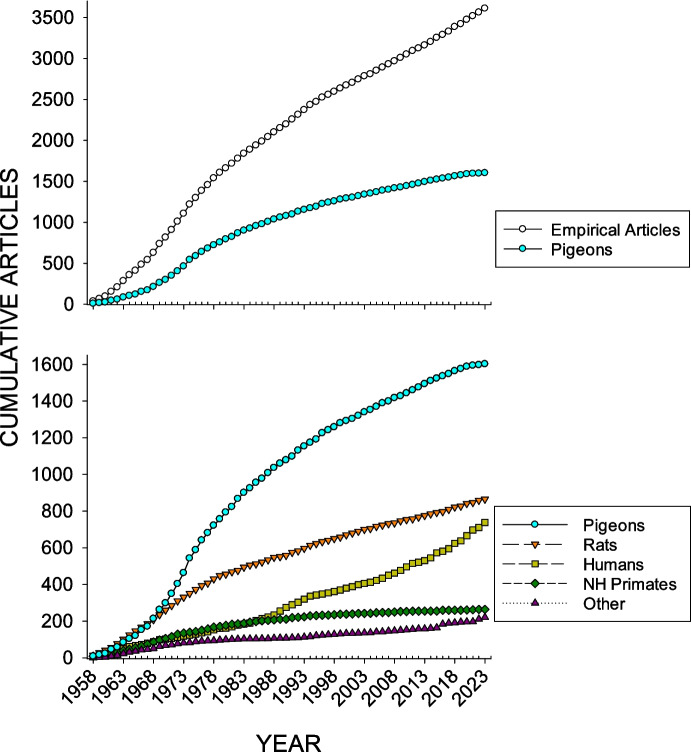
Cumulative Count of Empirical Articles and Pigeon JEAB Publications (Top Graph), as Well as the Cumulative Count of Articles per Each Taxonomic Category (Bottom Graph). *Note.* The x-axis shows the year-to-year values, 1958–2023

Figure 3 shows over the period 1958–2023 cumulative counts of articles in which pigeons were the research subjects compared to articles reporting empirical research based on rats, humans, nonhuman primates, and other species.

For the first 10 years of the journal’s existence (1958–1967), rats maintained the highest *percentage* of use in *JEAB* publications (i.e., the highest cumulative count). For the remainder of the journal’s time to the present (1968–2023), pigeons have maintained the highest cumulative count, with the highest percentage reached in 1987, at 49.4% (1008 of 2042 publications). By the end of 2023, pigeons were used in 1,602 of 3,611 *JEAB* empirical articles (44.4%, see Table 1).

**Table 1:** Subjects in JEAB Publications (1958–2023)

Subjects	Count (Articles)	Percent
*Total*	3,611	100%
*Pigeons*	1,602	44.4%
*Rats*	865	24.0%
*Humans*	738	20.4%
*Non-human Primates*	264	7.3%
*Other Species*	204	5.6%

The sum of each subject category adds to more than the total article count (as well as 100%), because more than one subject type could appear in an article

Although not the focus of this review, as of 2011 pigeons had appeared as subjects in only two reports in *JEABs* sister journal, the flagship *Journal of Applied Behavior Analysis* (Edwards & Poling, 2011), which is dedicated to publishing research relevant to the applications of behavior principles to problems of social significance. One of the reports was concerned with say-do correspondence (Lattal & Doepke, 2001) and the other on fluency (Poling et al., 2009). In addition, Kelley et al. (2015) published a *JABA* article in which they reported on renewal effects in both pigeons and children.

## Research with Pigeons beyond the Experimental Analysis of Behavior

A critical feature for behavior analysis has been to study and understand behavior as a subject matter in its own right (Skinner, 1957). Laboratory studies of pigeons and other subjects used in EAB research was assumed to generalize across species and settings, creating basic behavioral principles that eventually would guide both applied research and practice (e.g., Baer et al., 1968). Skinner’s own writings reflected this desire to see greater use of behavioral principles derived from basic research, including their application to society as a whole (Skinner, 1971, 1974, 1978). As such, it is valuable to document how behavioral research with laboratory pigeons has influenced overall scientific efforts, including endeavors that extend beyond the experimental analysis of behavior, both in terms of research and practice.

To examine the influence of behavioral research with pigeons on other scientific fields, a Web of Science search was conducted (March 12, 2024) using the search strategy pigeon* AND (behavior* OR behaviour*), netting 6,721 results published in 1980 or later. The Clarivate and InCite Citation Topics tools were used to organize the results, which are based on citation relationships and not the content or subject matter of their constituent documents. “Analyze Results” in the filter panel then was used to generate a tree map of the top 15 citation topics at the meso (medium or group) level of analysis. The results appear in Table 2.

**Table 2:** Web of Science Search Results for Pigeon Behavior

Field	Count (Articles)	Percentage
*Total*	6,721	100%
*Autism & Developmental Disorders*	2,292	34.1%
*Neuroscience*	1,181	17.5%
*Neuroscanning*	617	9.2%
*Zoology & Animal Ecology*	558	8.3%
*Biophotonics & Electromagnetic Field Safety*	353	5.3%
*Music*	176	2.6%
*Sleep Science & Circadian Systems*	125	1.9%
*Anesthesiology*	94	1.4%
*Entomology*	68	1.0%
*Dairy & Animal Sciences*	54	0.8%
*Bacteriology*	49	0.7%
*Hearing Loss*	47	0.7%
*Modeling & Simulation*	43	0.6%
*Substance Abuse*	38	0.6%
*Social Psychology*	34	0.5%

Only the top 15 categories are listed and therefore will not add up to the total count or 100%

This search was simplified to maximize links between behavioral research with pigeons and its influence on broader scientific fields. The research topic of autism and developmental disabilities was the top result, comprising just over one third (2,292 hits; 34.1%) of all results, probably because these are the two most studied and practiced areas of applied behavior analysis (ABA; Fisher et al., 2021; Foxx, 2008), with ABA as a field historically deeply connected to EAB (Branch & Malagodi, 1980; Mace & Critchfield, 2010; Rader et al., 2021).

Other areas of interest were fields that fall under or are related to the broader behavioral science subdisciplines such as neuroscience, neuroscanning, and circadian systems. These areas traditionally have used many conditioning procedures to investigate behavioral phenomena (Pierce & Cheney, 2017; Timberlake et al., 2005). In addition, the broader influence of laboratory pigeon research on comparative psychology is reflected in the category “Zoology & Animal Ecology,” which received the fourth highest count (553; 8.3%). This may be due in part to the impact of pigeon research on applied animal behavior efforts, including the animal training practices briefly noted in the introduction (see Lattal & Fernandez, 2022; for a review, see Fernandez & Martin, 2021).

Finally, some of the more remote links include the animal sciences and other similar production animal research, which have used laboratory operant conditioning techniques such as progressive-ratio schedules to study phenomena such as stimulus preferences (Patterson-Kane et al., 2008). Production animal science and applications occasionally rely on behavioral principles learned from laboratory experiments, as reflected in a recent study in which cows were trained to urinate in specific areas in an effort to reduce their environmental impact (Dirksen et al., 2021).

## The Pigeon’s Past and Future as a Subject

This review provided a brief history of the laboratory pigeon, including the antecedent events that facilitated the adoption of pigeons as laboratory subjects (e.g., domestication), and the impact of pigeons on behavioral research and application. Part of this impact is seen in the material detailed in the second and third sections above, through quantitative data on the frequency of pigeon use in research published in *JEAB,* and the number of articles connected to a Web of Science “pigeon” and “behavior” search terms.

This quantitative assessment of pigeon use in behavioral research complement and extend several earlier such surveys. Unlike these previous subject-use surveys, however, the present one provides a year-by-year analysis. That analysis verifies the trend of increasing use of pigeons between 1958 and 1982 documented in each of the earlier surveys (Grossett et al., 1982; Sokolowski et al., 2010; Zimmerman et al., 2015) followed by a decline in pigeon subjects beginning in the mid-1980s and continuing through 2015 (Sokolowski et al., 2010; Zimmerman et al., 2015). The present results extend this downward trend in pigeon use as *JEAB* subjects for almost another decade.

Many factors helped shape the initial selection of pigeons as laboratory subjects, including their long history of domestication and more recent use in Skinner’s earliest applications of behavioral principles outside of the laboratory. Within the experimental analysis of behavior, and particularly within *JEAB* articles, pigeons quickly rose to become the most frequently used research subject, even today accounting for nearly half of all subjects in over 50 years of all *JEAB* research articles. This reliance on pigeons as research subjects also has affected their influence in other fields, including other psychological sciences and applications (e.g., neurosciences; animal training and behavior).

Several factors may have contributed to the decline in recent years of the absolute number of experiments in which pigeons have been the subjects. Over the years, changes in federal regulations in the United States concerning the maintenance and care of experimental animals generally has added significantly to their maintenance costs. These costs are reflected in the additional infrastructure and bureaucracy needed to administer and oversee animal research programs as well as ever-increasing direct costs like the per diem fees for animal maintenance. A related variable is the diminished federal support for research with pigeons as subjects. Moreover, there has been a general decline in university-level support for animal research as more universities reorganize their program and funding priorities in the present period of budget austerity.

The absolute and proportional rise in human research, as shown in the cumulative counts in Fig. 3, may be either a cause or an effect (or both) of the shrinking populations of pigeons in EAB research. It also is the case that the research focus of EAB is shifting to problems for which humans are more appropriate participants. Research on stimulus equivalence, pioneered by Sidman and Tailby (1982), and hypothetical delay discounting tasks also account for some of the increased use of human participants. Furthermore, the rise of translational research in *JEAB*, with its own dedicated associate editor in the journal’s organizational structure, may be another source of a general shifting away from basic research with nonhuman animals. Human research certainly has its own methodological concerns and limitations (e.g., Perone et al., 1988); however, the present data suggest that using humans as research participants is rapidly expanding to either complement or replace the formerly ubiquitous pigeon as *JEAB’s* subject/participant of choice.

Although the use of pigeons as research subjects may be diminishing, their prominent role within the history of EAB and all of the behavioral sciences is well-established. Understanding the historical and continued impact of pigeons discussed in this review, behavior analysts hopefully can be more informed about how the humble pigeon in a box has contributed to the shaping of the science of behavior.

## Data Availability

Data is available upon request from the first author.
